# Characterization of the Fungal Microbiome (Mycobiome) in Fecal Samples from Dogs

**DOI:** 10.1155/2013/658373

**Published:** 2013-04-23

**Authors:** M. Lauren Foster, Scot E. Dowd, Christine Stephenson, Jörg M. Steiner, Jan S. Suchodolski

**Affiliations:** ^1^Gastrointestinal Laboratory, Department of Small Animal Clinical Sciences, College of Veterinary Medicine & Biomedical Sciences, Texas A&M University, College Station, TX 77843, USA; ^2^Molecular Research DNA Laboratory, Shallowater, TX 79363, USA

## Abstract

The prevalence and phylogenetic description of fungal organisms and their role as part of the intestinal ecosystem have not yet been studied extensively in dogs. This study evaluated the fungal microbiome of 19 dogs (12 healthy dogs and 7 dogs with acute diarrhea) using fungal tag-encoded FLX-Titanium amplicon pyrosequencing. Five distinct fungal phyla were identified, with *Ascomycota* (medians: 97.9% of obtained sequences in healthy dogs and 98.2% in diseased dogs) and *Basidiomycota* (median 1.0% in healthy dogs and median 0.5% in diseased dogs) being the most abundant fungal phyla. A total of 219 fungal genera were identified across all 19 dogs with a median (range) of 28 (4–69) genera per sample. *Candida* was the most abundant genus found in both the diseased dogs (median: 1.9%, range: 0.2%–38.5% of sequences) and healthy dogs (median: 5.2%, range: 0.0%–63.1% of sequences). *Candida natalensis* was the most frequently identified species. No significant differences were observed in the relative proportions of fungal communities between healthy and diseased dogs. In conclusion, fecal samples of healthy dogs and dogs with acute diarrhea harbor various fungal genera, and their role in gastrointestinal health and disease warrants further studies.

## 1. Introduction

Recent molecular-phylogenetic studies have revealed diverse microbial communities in the canine gastrointestinal (GI) tract and have emphasized the importance of the intestinal microbiota for gastrointestinal health [1]. The intestinal microbiota plays a vital role in the health of the GI tract, participates in the development of the host immune system, and also provides protection from invading pathogens [2]. Several studies have characterized the bacterial communities in the canine GI tract in health and disease [3–5]. However, limited information is available about the prevalence and classification of other members of the intestinal microbiome, such as fungal organisms. Previous studies that have described fungal organisms in the canine GI tract have either used culture based methods [6, 7], have utilized molecular-phylogenetic methods on pooled intestinal samples [8, 9], or have analyzed only a limited number of fungal sequences [10]. Studies in humans have suggested that the fungal microbiome may play a role in chronic GI disorders [11, 12]. Therefore, a more detailed description of the fungal microbiome (mycobiome) is needed to better understand the role of fungi in the GI tract of healthy dogs and dogs with GI disease.

The aim of this study was to describe the fungal communities present in fecal samples obtained from healthy dogs and dogs with acute, nonhemorrhagic diarrhea using high-throughput 18S rRNA gene pyrosequencing.

## 2. Materials and Methods

### 2.1. Fecal Samples

Naturally passed fecal samples were collected from a total of 19 privately owned dogs (12 healthy dogs and 7 dogs with acute diarrhea). All dogs, healthy and diseased, lived in Texas at the time of sample collection. The dogs of the healthy dog group had a median weight of 16.5 kg with a range of 2.6–35.0 kg and a median age of 5.6 years with a range of 2.0–15.0 years. The dogs of the diseased dog group had a median weight of 16.0 kg with a range of 2.5–28.0 kg and a median age of 7.0 years with a range of 1.0–15.0 years. The healthy dogs were owned by students and staff of Texas A&M University. At the time of sample collection, all 12 healthy dogs (Table 1) were free from any clinical signs of disease. A complete blood count and serum biochemistry profile were analyzed at the Texas Veterinary Medical Diagnostic Laboratory (College Station, TX). To exclude subclinical gastrointestinal or pancreatic disease in the healthy dogs, serum concentrations of cobalamin and folate (Immulite 2000, Siemens Healthcare Diagnostics Inc., Deerfield, IL, USA), pancreatic lipase immunoreactivity (cPLI; Spec cPL ELISA kit, IDEXX Laboratories, Westbrook, ME, USA), and trypsin-like immunoreactivity (cTLI; I-RIA kit, Siemens Medical Solution Diagnostics, Los Angeles, CA, USA) were measured at the Gastrointestinal Laboratory at Texas A&M University. Fecal samples from all dogs in the diseased and healthy groups were analyzed for *Clostridium perfringens* and *Clostridium difficile *using an enzyme-linked immunosorbent assay kit (*C. perfringens* Enterotoxin Test and *C. difficile* Enterotoxin Test, TechLab, Inc., Blacksburg, VA, USA), *Giardia* and *Cryptosporidium* using an indirect fluorescent antibody test kit (Merifluor, Meridian Bioscience Inc., Cincinnati, OH), *Campylobacter* spp. using PCR, and other pathogens using a routine fecal flotation.

One fecal sample per animal was collected immediately after natural defecation, stored at 4°C before and during transport to the laboratory (within 24 hours of sample collection), and subsequently stored frozen at −80°C until DNA extraction.

For the diseased dogs, leftover fecal samples that were submitted from veterinary hospitals in the College Station/Houston area for fecal pathogen analysis unrelated to the current study were analyzed. Questionnaires were sent to submitting veterinarians to enquire about the clinical signs of gastrointestinal disease, the duration, and the final diagnosis. Furthermore, to ensure that samples were handled in a similar fashion as the samples from the healthy dogs, information was obtained about the collection, storage, and shipping of samples to the laboratory. Only samples that were handled in a similar fashion to the samples from healthy dogs were analyzed. Leftover fecal samples for the diseased dogs (Table 1) were chosen based on a presenting complaint of acute, uncomplicated, nonhemorrhagic diarrhea (defined as <72 hours of onset) that resolved with symptomatic treatment.

At the time of sample collection, none of the evaluated healthy and diseased dogs were receiving any medications expected to alter the gut microbiota (i.e., antibiotics) and were vaccinated and dewormed regularly. All dogs were fed commercial diets and no diet change was reported within the 3-week period prior to sample collection.

This study was approved by the Clinical Research Review Committee of Texas A&M University (CRRC#07-38).

### 2.2. DNA Extraction

Genomic DNA was extracted from each fecal sample using a bead-beating technique followed by phenol-chloroform-isoamyl alcohol extraction as described previously [13].

### 2.3. 18S rRNA Gene Pyrosequencing for Fungal Organisms (fTEFAP)

Tag-encoded FLX-Titanium amplicon pyrosequencing (fTEFAP) and data processing for fungal organisms were performed as described previously [8, 14] with panfungal primers forward funSSUF-TGGAGGGCAAGTCTGGTG and reverse funSSUR-TCGGCATAGTTTATGGTTAAG.

The raw data from fTEFAP was screened and trimmed based on quality scores (nominal phred20), binned into individual sample collections, and then depleted of any chimeras using B2C2 (http://www.researchandtesting.com/B2C2.html) [15]. The sequences were then compared against a curated fungal sequence database as reported previously [8, 14]. Fungal sequences were grouped into operational taxonomic units (OTUs) based on identity scores to known 18S fungal sequences; >97% of identity were reported at the species level, between 95% and 97% at the genus level, between 90% and 95% at the family level, and between 80% and 90% at the order level.

### 2.4. Statistical Analyses

The pyrosequencing results were expressed as a percentage of the total fungal community in each dog. Only taxa that were present in at least 50% of dogs (either healthy or diseased) were included in the analysis. All percentage data was tested for normality using the Kolmogorov-Smirnov normality test. As the data was found to be nonparametric, a Mann-Whitney *U* test was used to compare the percentages of fungal organisms between the healthy and the diseased dogs at the phylum, class, order, family, and genus levels. All statistical analyses were conducted using a commercially available statistical software program (Prism 5, GraphPad, San Diego, CA, USA). Significance level was set at *P* < 0.05 for all comparisons.

To visualize differences in the relative abundance of fungal genera in individual samples as a heat map, a double dendrogram was generated using multivariate hierarchical clustering methods based upon Furthest Neighbor metric with Euclidean distances in NCSS 2007 (NCSS, Kaysville, Utah) [16]. The sequencing coverage for each sample was calculated according to Good using the formula [1 − (*n*/*N*)] × 100, where *n* is the number of unique sequences and *N* is the total number of sequences obtained for each sample.

## 3. Results

### 3.1. Animals

The results of the serum biochemistry profile, complete blood count, serum concentrations of cobalamin, folate, cPLI, and cTLI did not reveal abnormalities in the healthy group. Review of the clinical records of the dogs with acute diarrhea showed that all dogs recovered uneventfully with symptomatic therapy. Results of fecal examination (i.e., fecal flotation, fecal analyses for *C. perfringens*, *C. difficile*, *Cryptosporidium*, and *Giardia*, and PCR analysis for *Campylobacter *spp.) on all dogs did not reveal the presence of any specific enteropathogen.

### 3.2. Pyrosequencing for Fungal Organisms

A total of 57,179 sequences (median 2800, range 840–6000 per sample) of good quality were obtained. To allow for equal sequencing depth across all samples, 840 randomly selected sequences were analyzed per sample, as described previously [16]. A total of five phyla were identified. Of these, *Ascomycota* and *Basidiomycota* were found in ≥50% of dogs in both groups of diseased and healthy dogs. The remaining phyla, *Chytridiomycota*, *Neocallimastigomycota*, and *Microsporidia,* were found in ≤50% of dogs in both groups. A total of 219 fungal genera were identified across all 19 dogs in this study with a median of 28 genera per dog and a range of 4–69 genera per dog. There were a median of 32 genera (range 10–55) per dog in the healthy group and a median of 18 genera (range 4–69) per dog in the diseased group. The mean (±SD) coverage was 0.956 (±0.0256) based on a sequencing depth of 840 sequences per samples.

No significant differences (*P* > 0.05) of the percentage of fungal organisms at the phylum, class, order, family, and genus levels were found between the healthy and diseased dogs. There were no significant differences in age (*P* = 0.983) or weight (*P* = 0.577) between the healthy and diseased groups.  Table 2 summarizes the relative percentages in terms of median and range for the most abundant fungal groups on the various phylogenetic levels based on pyrosequencing results (phylum, class, order, and genus levels). Only groups that were present in at least 50% of either healthy or diseased dogs are shown.

Members of *Ascomycota* were found in all 19 dogs and this was the most abundant fungal phylum in both the healthy dogs and the diseased dogs with a median of 97.90% and a range of 63.2%–100.0% and a median of 91.4% and a range of 91.4%–100.0% of all fungal sequences, respectively. *Basidiomycota* was the second most abundant fungal phylum with a median of 1.0% and a range of 0.0–36.8% for the healthy group and a median of 0.5% and a range of 0.0%–7.8% for the diseased group. *Basidiomycota* was found in eight healthy dogs and five diseased dogs.

Within *Ascomycota*, classes in either the healthy or the diseased groups consisted of *Dothideomycetes*, *Saccharomycetes*, *Eurotiomycetes*, *Taphrinomycetes*, and *Sordariomycetes*, with *Dothideomycetes* being the most abundant class (median: 34.0%, range: 0.3%–74.4% for healthy dogs; median: 28.8%, range: 19.1%–90.9% for diseased dogs). The most prominent orders in *Dothideomycetes* were *Pleosporales* (median: 16.1%, range: 0.0%–50.9% for healthy dogs and a median of 15.0% and a range of 0.0%–81.4% for diseased dogs) and *Capnodiales* (median: 11.5%, range: <0.1%–36.0% for healthy dogs; median: 8.7%, range: 0.8%–81.8% for diseased dogs). *Pleosporales* was the most diverse of the two orders, containing four genera and eight species.

Within *Basidiomycota,* the predominant classes were *Agaricomycetes* and *Ustilaginomycetes*, with the latter consisting almost exclusively the orders *Ustilaginales* and the genus *Ustilaginaceae*.

**Figure 1:**
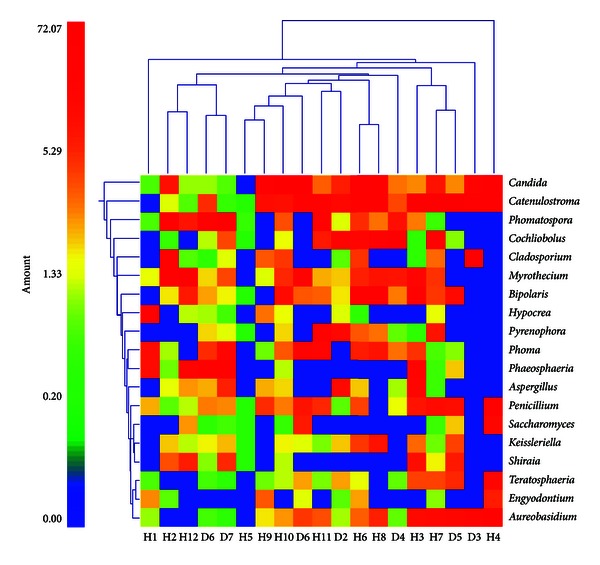
Dual hierarchal dendrogram based upon the predominant fungal genera. The clustering is based upon Furthest Neighbor metric with Euclidean distances. The heat map represents the relative percentages of the most abundant fungal genera identified in each sample (H = healthy, D = diseased).

Table 3 shows several species in the major fungal groups identified in the dog feces using pyrosequencing, as well as some species considered to have the potential to be pathogenic. The sequences were blasted against the NCBI database. The accession number, the similarity to their closest neighbor in the NCBI, and the number of healthy and diseased dogs harboring these species are summarized in this table.  Figure 1 shows the differences in the relative abundance of the fungal genera in each dog displayed as a heat map.

## 4. Discussion

In this study, 454-pyrosequencing of the fungal 18S rRNA gene was utilized to characterize the fungal microbiome present in canine feces. Fecal samples from a total of 19 dogs were individually evaluated. Seven dogs were classified as diseased based on the presence of acute nonhemorrhagic diarrhea, and the remaining 12 were classified as healthy. In the current study, no significant differences in the relative proportions of fungal groups were observed between the diseased and healthy dogs. The most abundant phylum in canine feces was *Ascomycota* (median: 97.9%, range: 63.2%–100.0% for healthy and median: 98.2%, range: 91.4%–100.0% for diseased dogs). The most abundant classes included *Dothideomycetes *and *Saccharomycetes, *with *Candida* found to be the most abundant genus.

Previous studies using either culture-based or molecular methods have provided some information about the fungal microbiome present in the GI tract of dogs [6–9, 17]. Based on cultivation studies, Mentula et al. observed a higher prevalence of yeast in the lumen of the jejunum compared to the feces of healthy dogs (27% versus 5%, resp.) [7]. Benno et al. reported the presence of yeasts and molds in the stomach, ileum, colon, and rectum in 2 of 8 healthy Beagle dogs [6]. However, no further specifics regarding the phylogenetic classification of these organisms were reported in these two studies. Using a panfungal PCR, Suchodolski et al. reported that 60% of healthy dogs and 76% of dogs with chronic enteropathies were positive for fungal DNA in small intestinal brush samples [10]. All 51 phylotypes identified by Suchodolski et al. were members of the phyla *Ascomycota *(32 phylotypes) or *Basidiomycota *(19 phylotypes) [10]. Handl et al. analyzed pooled fecal samples obtained from 12 healthy dogs using 18S rRNA gene pyrosequencing and found that *Ascomycota* comprised 99% of the fungal sequences, with *Saccharomycetes* found to be the most abundant class at 85% and *Candida* found to be the most abundant genus [8]. Similarly, using a shotgun DNA sequencing approach, Swanson et al. also identified *Ascomycota* and *Basidiomycota* as the most abundant phyla in pooled fecal samples of 6 healthy dogs [9]. However, the overall abundance of fungal sequences was low (0.01% of the canine metagenome) and only 3 distinct phylotypes were identified, most likely due to an insufficient sequencing depth [9].

Our results are in general agreement with the previously cited studies. By analyzing the fungal 18S rRNA gene in individual fecal samples, we identified a higher number of fungal OTUs (at 97% similarity; OTU_97_) compared to a previous study that used a metagenomic approach based on DNA shotgun sequencing of fecal samples and which reported only 3 OTUs [9]. Also, our study revealed a higher number of OTUs than reported for pooled fecal samples (i.e., 33 OTU_97_). This study also suggests that the fecal mycobiome harbors more fungal OTUs compared to the small intestine, as one study examining the small intestinal fungal microbiome reported that 79% of dogs positive for fungal DNA harbored only one unique phylotype [10]. However, the species richness of fungal organisms appears to be lower compared to the bacterial richness, as several hundred bacterial OTUs have been reported [8] in fecal samples of dogs.

While this study provided insight into the diversity of the fungal microbiome in healthy dogs and dogs with acute diarrhea, some limitations need to be noted. The methods employed did not allow quantitative determination of the fungal abundance. Previous metagenomic studies performed on canine fecal samples suggest that fungal sequences make up a small proportion of the total fecal microbiota with 0.3% of obtained sequences [9]. Quantitative studies utilizing fluorescence *in situ* hybridization (FISH) of fungal small-subunit rRNA probes estimated the abundance of fungi as <2% in fecal samples from mice [18] and <0.03% in fecal samples from humans [11]. Future studies require the use of a more quantitative enumeration technique such as FISH to quantify the abundance of fungi in feces of dogs. The current study was limited in terms of the number of animals and the comparison of a healthy community to a community with only one disease type, and these results were obtained from fecal samples only, not biopsies. The current cost of high-throughput sequencing prohibited the use of a larger sample size.

In conclusion, the current study provides information about the fungal microbiome present in canine feces from both healthy dogs and dogs with acute, nonhemorrhagic diarrhea. The results provide a baseline for future studies evaluating the fungal microbiome in dogs with various gastrointestinal disorders.

## Figures and Tables

**Table 1:** Dogs enrolled into this study.

ID	Health status	Age	Breed	Sex	Weight (kg)
H1	Healthy	4.2	Shih-tzu	fs	7.0
H2	Healthy	4.0	English Bulldog	fs	20.7
H3	Healthy	15.0	Chihuahua	fs	2.6
H4	Healthy	2.0	Golden Retriever	mn	35.0
H5	Healthy	7.8	Mixed breed	mn	30.4
H6	Healthy	4.0	Dachshund	fs	6.0
H7	Healthy	9.6	Mixed breed	fs	32.1
H8	Healthy	10.0	Mixed breed	mn	28.0
H9	Healthy	3.5	Miniature Schnauzer	mn	9.5
H10	Healthy	5.2	Terrier Mix	mn	27.7
H11	Healthy	5.9	Brussels Griffon	mn	6.4
H12	Healthy	9.2	Beagle	mn	12.4
D1	Diseased	1.5	Labrador Retriever	fs	28.0
D2	Diseased	11.0	Mixed Breed	fs	14.5
D3	Diseased	15.0	Cocker Spaniel	mn	16.0
D4	Diseased	7.0	Chihuahua	fs	2.5
D5	Diseased	2.3	King Charles	fs	6.0
D6	Diseased	14.0	Golden Retriever	fs	24.8
D7	Diseased	1.0	Labrador mix	mn	18.0

m: male intact; f: female intact; mn: male neutered; fs: female spayed.

**Table 2:** Relative percentages of the most abundant fungal groups on the various phylogenetic levels based on pyrosequencing. The letters in parenthesis indicate phylogenetic levels (p: phylum, c: class, o: order, f: family, g: genus).

Taxonomy	Range (minimum %–maximum %) and medians (%)					Number of dogs harboring taxa	
	Healthy range	Healthy median	Diseased range	Diseased median	Mann-Whitney *P* value	Healthy	Diseased
Ascomycota (p)	63.20–100.00	97.90	91.40–100.00	98.20	0.58	12	7
*Catenulostroma *(g)	0.00–31.41	8.23	0.09–48.36	7.09	0.83	11	7
*Dothideomycetes *(c)	0.26–74.38	34.03	19.07–90.91	28.82	0.29	12	7
*Capnodiales *(o)	0.04–36.00	11.45	0.84–81.80	8.65	0.97	12	7
*Davidiellaceae *(f)	0.00–29.04	0.13	0.00–63.64	0.06	0.79	7	4
*Cladosporium *(g)	0.00–29.04	0.13	0.00–63.64	0.06	0.79	7	4
*Schizothyriaceae *(f)	0.04–33.96	8.55	0.14–51.64	8.42	0.83	12	7
*Pleosporales *(c)	0.00–50.88	16.09	0.00–81.38	14.96	0.80	11	6
*Phoma *(g)	0.00–8.27	1.28	0.00–9.09	1.86	0.83	9	5
*Massarinaceae *(f)	0.00–5.26	0.47	0.00–2.54	0.80	0.83	9	5
*Keissleriella *(g)	0.00–5.26	0.47	0.00–2.54	0.80	0.83	9	5
*Phaeosphaeriaceae *(f)	0.00–9.22	0.06	0.00–9.73	0.00	0.82	6	3
*Teratosphaeria *(g)	0.00–7.14	0.32	0.00–3.27	0.26	0.90	8	6
*Pleosporaceae *(f)	0.00–43.86	6.09	0.00–80.59	6.90	0.97	10	6
*Cochliobolus *(g)	0.00–21.05	0.09	0.00–72.07	0.60	0.64	8	5
*Phaeosphaeria *(g)	0.00–9.22	0.06	0.00–9.73	0.00	0.82	6	3
*Pyrenophora *(g)	0.00–11.92	0.05	0.00–7.09	0.26	0.93	7	4
*Bipolaris *(g)	0.00–21.05	2.39	0.00–6.54	1.41	0.55	9	6
*Shiraiaceae *(f)	0.00–5.82	0.02	0.00–4.36	0.00	0.89	6	3
*Shiraia *(g)	0.00–5.82	0.02	0.00–4.36	0.00	0.89	6	3
*Dothideales *(o)	0.00–10.71	1.71	0.04–9.09	0.44	0.97	9	7
*Dothioraceae *(f)	0.00–10.71	1.71	0.04–9.09	0.44	0.97	9	7
*Aureobasidium *(g)	0.00–10.7	1.71	0.05–9.09	0.44	0.97	9	7
*Saccharomycetes *(c)	0.19–99.40	10.94	0.35–42.66	6.87	0.58	12	7
*Saccharomycetales *(o)	0.18–99.40	10.94	0.34–42.66	6.87	0.58	12	7
*Wickerhamomycetaceae* (f)	0.00–63.08	5.20	0.19–38.46	1.86	0.53	11	7
*Saccharomycetaceae *(f)	0.00–99.4	0.67	0.00–14.91	0.14	0.73	9	4
*Saccharomyces *(g)	0.00–7.14	0.00	0.00–4.19	0.06	0.52	5	4
*Candida *(g)	0.00–63.08	5.20	0.19–38.46	1.86	0.53	11	7
*Eurotiomycetes *(c)	0.04–14.7	4.16	0.00–43.9	4.90	0.97	12	6
*Eurotiales *(o)	0.04–14.29	3.00	0.00–10.64	4.89	0.97	12	6
*Trichocomaceae *(f)	0.04–14.29	3.00	0.00–10.64	4.89	0.90	12	6
*Aspergillus *(g)	0.00–7.28	0.42	0.00–6.65	0.53	0.86	7	4
*Penicillium *(g)	0.00–14.29	2.09	0.00–5.81	1.53	0.74	11	6
*Taphrinomycetes *(c)	0.00–30.53	4.49	0.00–11.76	5.05	0.61	11	6
*Taphrinales *(o)	0.00–30.53	4.49	0.00–11.76	5.05	0.61	11	6
*Taphrinaceae *(f)	0.00–30.53	3.69	0.00–11.76	5.05	0.74	11	6
*Sordariomycetes *(o)	0.00–52.32	9.84	0.00–61.46	10.37	0.97	11	5
*Phomatospora *(g)	0.00–17.91	1.67	0.00–43.13	0.66	0.97	10	4
*Meliolales *(o)	0.00–17.91	1.67	0.00–43.13	0.66	0.97	10	4
*Xylariales *(o)	0.00–1.55	0.00	0.00–5.59	0.44	0.17	4	4
*Xylariaceae *(f)	0.00–1.55	0.00	0.00–5.59	0.25	0.17	3	4
*Sordariales *(o)	0.00–17.89	0.03	0.00–0.44	0.00	0.54	6	2
*Hypocreales *(o)	0.00–30.53	4.49	0.00–9.09	2.71	0.10	11	5
*Ophiocordycipitaceae *(f)	0.00–32.58	3.73	0.00–9.09	1.99	0.33	11	5
*Hypocreaceae *(f)	0.00–17.33	0.03	0.00–0.66	0.00	0.49	6	3
*Hypocrea *(g)	0.00–17.33	0.03	0.00–0.66	0.00	0.49	6	3
*Myrothecium *(g)	0.00–32.58	3.25	0.00–9.09	1.10	0.47	10	5
*Clavicipitaceae *(f)	0.00–1.86	0.21	0.00–0.66	0.00	0.36	8	3
*Cordycipitaceae *(f)	0.00–0.61	0.00	0.00–0.09	0.00	0.51	3	1
*Engyodontium *(g)	0.00–3.57	0.11	0.00–0.69	0.00	0.24	6	2
*Papulosaceae *(f)	0.00–17.9	1.67	0.00–43.10	0.67	0.97	12	6
Basidiomycota (p)	0.00–36.75	0.97	0.00–7.76	0.54	0.97	8	5
*Agaricomycetes *(c)	0.00–3.31	0.00	0.00–4.88	0.27	0.23	5	5
*Ustilaginomycetes *(c)	0.00–33.44	0.15	0.00–2.88	0.00	0.12	7	1
*Ustilaginales *(o)	0.00–24.83	0.05	0.00–0.66	0.00	0.13	6	1
*Ustilaginaceae *(f)	0.00–24.83	0.05	0.00–0.66	0.00	0.13	6	1

**Table 3:** Fungal species identified in feces from dogs using pyrosequencing. The accession number and similarity to their closest relative in the NCBI database and the number of healthy and diseased dogs harboring the species are summarized.

Fungal species	Accession no. to closest relative	Similarity	Healthy dogs	Diseased dogs
*Catenulostroma abietis *	FJ267703	99	11	7
*Bipolaris eleusines *	DQ337382	99	0	1
*Bipolaris sorokiniana *	DQ337383	99	8	6
*Candida albicans *	AF114470	99	0	1
*Candida austromarina *	AB013560	99	3	1
*Candida castellii *	AY497752	92	2	1
*Candida glycerinogenes *	AY584809	99	2	0
*Candida homilentoma *	AB018166	99	1	0
*Candida khmerensis *	AB158655	98	1	0
*Candida mesenterica *	AB013552	99	1	0
*Candida natalensis *	AB013541	99	10	6
*Candida neerlandica *	EF120593	99	2	0
*Candida zeylanoides *	EU590665	99	1	0
*Aspergillus flavipes *	AB002061	98	0	1
*Aspergillus niger *	JX112703	99	4	3
*Aspergillus ochraceus *	AB008405	99	2	1
*Aspergillus penicillioides *	AB002078	99	4	0
*Aspergillus terreus *	JN639854	99	5	2
*Penicillium brevicompactum *	AF548082	99	4	0
*Penicillium charlesii *	FJ430768	99	4	1
*Penicillium commune *	EU263609	99	2	1
*Penicillium coprobium *	FJ430772	99	1	0
*Penicillium janthinellum *	AB293968	100	7	4
*Penicillium tardum *	AF245233	99	4	1
*Penicillium verruculosum *	AF510496	100	4	2
*Myrothecium cinctum *	AJ301996	99	6	5
*Myrothecium gramineum *	FJ825369	99	8	3
*Myrothecium leucotrichum *	AJ301992	99	4	2
*Cryptococcus gastricus *	DQ645513	98	2	0
*Cryptococcus surugaensis *	AB100440	97	1	0

## References

[1] Suchodolski JS (2011). Companion animals symposium: microbes and gastrointestinal health of dogs and cats. *Journal of Animal Science*.

[2] Neish AS (2009). Microbes in gastrointestinal health and disease. *Gastroenterology*.

[3] Suchodolski JS, Garcia-Mazcorro JF, Unterer S (2012). The fecal microbiome in dogs with acute diarrhea and idiopathic inflammatory bowel disease. *PLoS ONE*.

[4] Garcia-Mazcorro JF, Suchodolski JS, Jones KR (2012). Effect of the proton pump inhibitor omeprazole on the gastrointestinal bacterial microbiota of healthy dogs. *FEMS Microbiology Ecology*.

[5] Bell JA, Kopper JJ, Turnbull JA, Barbu NI, Murphy AJ, Mansfield LS (2008). Ecological characterization of the colonic microbiota of normal and diarrheic dogs. *Interdisciplinary Perspectives on Infectious Diseases*.

[6] Benno Y, Nakao H, Uchida K, Mitsuoka T (1992). Impact of the advances in age on the gastrointestinal microflora of beagle dogs. *The Journal of Veterinary Medical Science*.

[7] Mentula S, Harmoinen J, Heikkilä M (2005). Comparison between cultured small-intestinal and fecal microbiotas in beagle dogs. *Applied and Environmental Microbiology*.

[8] Handl S, Dowd SE, Garcia-Mazcorro JF, Steiner JM, Suchodolski JS (2011). Massive parallel 16S rRNA gene pyrosequencing reveals highly diverse fecal bacterial and fungal communities in healthy dogs and cats. *FEMS Microbiology Ecology*.

[9] Swanson KS, Dowd SE, Suchodolski JS (2011). Phylogenetic and gene-centric metagenomics of the canine intestinal microbiome reveals similarities with humans and mice. *The ISME Journal*.

[10] Suchodolski JS, Morris EK, Allenspach K (2008). Prevalence and identification of fungal DNA in the small intestine of healthy dogs and dogs with chronic enteropathies. *Veterinary Microbiology*.

[11] Ott SJ, Kühbacher T, Musfeldt M (2008). Fungi and inflammatory bowel diseases: alterations of composition and diversity. *Scandinavian Journal of Gastroenterology*.

[12] Kühbacher T, Ott SJ, Helwig U (2006). Bacterial and fungal microbiota in relation to probiotic therapy (VSL#3) in pouchitis. *Gut*.

[13] Suchodolski JS, Ruaux CG, Steiner JM, Fetz K, Williams DA (2004). Application of molecular fingerprinting for qualitative assessment of small-intestinal bacterial diversity in dogs. *Journal of Clinical Microbiology*.

[14] Lucero ME, Unc A, Cooke P, Dowd S, Sun S (2011). Endophyte microbiome diversity in micropropagated *Atriplex canescens* and *Atriplex torreyi* var *griffithsii*. *PLoS ONE*.

[15] Gontcharova V, Youn E, Wolcott RD, Hollister EB, Gentry TJ, Dowd S (2010). Black box chimera check (B2C2): a windows-based software for batch depletion of chimeras from bacterial 16S rRNA gene datasets. *The Open Microbiology Journal*.

[16] Suchodolski JS, Dowd SE, Wilke V, Steiner JM, Jergens AE (2012). 16S rRNA gene pyrosequencing reveals bacterial dysbiosis in the duodenum of dogs with idiopathic inflammatory bowel disease. *PLoS ONE*.

[17] Suchodolski JS, Camacho J, Steiner JM (2008). Analysis of bacterial diversity in the canine duodenum, jejunum, ileum, and colon by comparative 16S rRNA gene analysis. *FEMS Microbiology Ecology*.

[18] Scupham AJ, Presley LL, Wei B (2006). Abundant and diverse fungal microbiota in the murine intestine. *Applied and Environmental Microbiology*.

